# Association of vitamin D supplementation with respiratory tract infection in infants

**DOI:** 10.1111/mcn.12987

**Published:** 2020-03-05

**Authors:** Miao Hong, Ting Xiong, Junmei Huang, Yuanjue Wu, Lixia Lin, Zhen Zhang, Li Huang, Duan Gao, Huanzhuo Wang, Chun Kang, Qin Gao, Xuefeng Yang, Nianhong Yang, Liping Hao

**Affiliations:** ^1^ Department of Nutrition and Food Hygiene, Hubei Key Laboratory of Food Nutrition and Safety and the Ministry of Education (MOE) Key Laboratory of Environment and Health, School of Public Health, Tongji Medical College Huazhong University of Science and Technology Wuhan China; ^2^ Department of Clinical Nutrition People's Hospital of Sanya City Sanya China

**Keywords:** feeding, infant, respiratory tract infection, vitamin D supplementation

## Abstract

Vitamin D deficiency has been reported to be associated with respiratory tract infection (RTI). However, evidence regarding the effects of vitamin D supplementation on susceptibility of infants to RTI is limited. In this prospective birth cohort study, we examined whether vitamin D supplementation reduced RTI risk in 2,244 infants completing the follow‐up from birth to 6 months of age. The outcome endpoint was the first episode of paediatrician‐diagnosed RTI or 6 months of age when no RTI event occurred. Infants receiving vitamin D supplements at a daily dose of 400–600 IU from birth to the outcome endpoint were defined as vitamin D supplementation and divided into four groups according to the average frequency of supplementation: 0, 1–2, 3–4, and 5–7 days/week. We evaluated the relationship between vitamin D supplementation and time to the first episode of RTI with Kaplan–Meier plots. The associations of vitamin D supplementation with infant RTI, lower RTI (LRTI), and RTI‐related hospitalization were assessed using modified Poisson regression. The median time to first RTI episode was 60 days after birth (95% CI [60, 90]) for infants without supplementation and longer than 6 months of age for infants with supplementation (*p* < .001). We observed inverse trends between supplementation frequency and risk of RTI, LRTI, and RTI‐related hospitalization (*p* for trend < .001), with the risk ratios in the 5–7 days/week supplementation group of 0.46 (95% CI [0.41, 0.50]), 0.17 (95% CI [0.13, 0.24]), and 0.18 (95% CI [0.12, 0.27]), respectively. These associations were significant and consistent in a subgroup analysis stratified by infant feeding.

**Key messages**

- Infants with vitamin D supplementation had a longer period of time without experiencing a respiratory tract infection episode compared with infants without supplementation.
- Routine vitamin D supplementation to infants was associated with a reduced risk of respiratory tract infection, lower respiratory tract infection, and hospitalization for respiratory tract infection during the first 6 months of life, with the lowest risk found in infants receiving supplements 5–7 days/week.
- The inverse association of vitamin D supplementation and respiratory tract infection was independent of infant feeding.

## 1 INTRODUCTION

Respiratory tract infections (RTIs) are the leading cause of morbidity and mortality in children younger than 5 years worldwide, with the highest rates occurring in the first year of life (Troeger et al., 2018). RTIs are also a major cause of hospitalization in children younger than 5 years, especially in infants whose rate of hospitalization was about 1.3 times higher than the overall rate in children aged 0–59 months, causing a substantial burden on health care and impacting early childhood development (Nair et al., 2013).

Vitamin D, an essential nutrient mainly synthesized in the skin by direct exposure to sunlight, first came into public sight for its important function in calcium metabolism and bone health (Gil, Plaza‐Diaz, & Mesa, 2018; Gröber, Spitz, Reichrath, Kisters, & Holick, 2014). Vitamin D deficiency, which commonly results from limited direct exposure to sunlight, the use of sunscreen protection, and inadequate nutritional intake of vitamin D from natural diet, is a global health problem among all age populations, especially in pregnant women and their newborns (Hilger et al., 2014; Holick, 2017; van Schoor & Lips, 2011; Yun et al., 2017). During infancy, due to poor vitamin D availability in breast milk (Við Streym et al., 2016; Wall et al., 2016) and limited sunlight exposure at this age, for many infants, supplementation is required to achieve vitamin D sufficiency (Högler, 2015).

Recently, emerging studies have linked an association between low levels of blood 25‐hydroxyvitamin D (25(OH)D) and increased susceptibility to RTI in early childhood (Binks, Smith Vaughan, Marsh, Chang, & Andrews, 2016; Camargo et al., 2011; Karatekin, Kaya, SalihogLu, Balci, & NuhogLu, 2009; Lai et al., 2017; Magnus et al., 2013; Mohamed & Al‐Shehri, 2012). In fact, vitamin D is assumed to have a regulatory role in fetal lung growth and immune system development (Bouillon et al., 2008; Christakos et al., 2013; Clancy et al., 2013; Gil et al., 2018; Lai et al., 2017; Prietl, Treiber, Pieber, & Amrein, 2013; Wei & Christakos, 2015), which underscores the promising important role of vitamin D in respiratory health.

In recent studies, researchers revealed a beneficial role of vitamin D supplementation in RTI prevention (Autier et al., 2017; Grant et al., 2015; Martineau et al., 2017). To date, current studies on the association of vitamin D supplementation with RTI among infants were limited. Given the high vulnerability to RTI during infancy (Troeger et al., 2018), the aim of this prospective cohort study was to determine whether routine vitamin D supplementation to infants can lower their susceptibility to RTI.

## 2 METHODS

### 2.1 Study design and data collection

We carried out a planned prospective cohort study to examine the association between vitamin D supplementation and RTI risk in infants, from the Tongji Maternal and Child Health Cohort study where the enrolment was conducted among pregnant women at a gestation period of 8–16 weeks from 2013 to 2016 in Wuhan, China. After signing the written informed consent, the pregnant women were invited to join a face‐to‐face interview administered by trained investigators with a structured questionnaire to obtain their sociodemographic characteristics, lifestyle behaviours, and health data at baseline. Detailed descriptions of the cohort have been published elsewhere (Wang et al., 2017; Wu et al., 2019; Zhong et al., 2017). After childbirth, the data on neonatal outcomes including gestational age, sex, birth date, and birthweight were obtained through face‐to‐face interviews within 72 hr of delivery in the maternity wards and from hospital medical records. During the postnatal 6 months, the mother–infant pairs were prospectively followed up with questionnaires by telephone interview administered by trained investigators at 1, 3, and 6 months post‐partum, to collect and update the infant feeding, illness, and supplement use.

At each telephone interview, parents were asked: (a) What are you currently feeding your baby? (b) On what date was your baby started on formula (if the baby was currently being mixed fed or formula fed on a regular or daily basis)? (c) How many times a day is your baby fed infant formula and how much formula milk does the baby usually consume at each feeding? (d) When was the last time the baby was breastfed (if the baby was currently being formula fed)?

Regarding respiratory illnesses, parents were asked: (a) Had your baby been taken to a hospital or clinic due to respiratory disease symptoms since the previous interview? (2) What was the date of each respiratory‐related hospital or clinic visit and what was the diagnosis given by the paediatrician? (3) Was the baby hospitalized for such respiratory‐related illness?

Regarding vitamin D supplementation, parents were asked: (a) Had the baby received vitamin D supplements? (b) On what date did the baby start to receive the supplementation? (c) Was the baby currently receiving the supplements? (d) On what date did the baby stop receiving supplementation? (e) How many days per week on average did the baby receive the supplement during the supplementation? (f) What was the brand name of the supplement?

### 2.2 Study population

The eligibility criteria for the present study were as follows: healthy singleton infants (no congenital abnormalities and underlying chronic disorders), gestational age at 37–42 weeks, birthweight at 2,500–4,000 g. In total, 5,099 mother–infant pairs were screened for eligibility. We further restricted the participants to 2,344 mother–infant pairs who completed 1‐, 3‐, and 6‐month follow‐up interviews, and we excluded 100 (4.2%) mother–infant pairs missing information on vitamin D supplementation or feeding or RTI outcome. After exclusions, there were a total of 2,244 mother–infant pairs identified for the final analysis (Figure S1).

### 2.3 Outcome endpoint and measures

The health outcome of interest was the first episode of RTI in the first 6 months of life. The outcome endpoint was the time of the first episode of RTI or the end of the follow‐up at 6 months of age (event occurred first as outcome endpoint—e.g., if no RTI event occurred in the first 6 months of life, then the end of the follow‐up at 6 months of age was considered as the outcome endpoint). We defined an infant RTI episode as the report by the mother of a baby having a paediatrician‐diagnosed common cold or bronchitis or bronchiolitis or pneumonia; then lower RTI (LRTI) referred to bronchitis, bronchiolitis, or pneumonia; and RTI‐related hospitalization referred to hospitalization due to RTI. The primary outcome measures in the study were the time to the first episode of RTI and the risk ratios (RRs) for associations of vitamin D supplementation with RTI, LRTI, and RTI‐related hospitalization, in the first 6 months of life.

### 2.4 Vitamin D supplementation and infant feeding

We designated infants receiving vitamin D supplements at a daily dose of 400–600 IU from birth to the outcome endpoint as exposure to vitamin D supplementation. Then, the average weekly frequency of vitamin D supplementation (days/week) was calculated for each infant by dividing the total number of days of supplementation by total number of weeks from birth to the endpoint. Based on the average frequency of vitamin D supplementation, we divided the infants into four groups: 0, 1–2, 3–4, and 5–7 days/week (Chao, Brunel, Faris, & Veugelers, 2013).

Considering the fortification of vitamin D in infant formula (10–25 IU/100 kJ, approximately 28–70 IU/100 ml; China Food and Drug Administration, 2017), we divided the infants into further subgroups, namely, the full breastfeeding and any formula feeding (mixed feeding or full formula feeding) subgroups. Full breastfeeding in our study was defined as feeding the infant with human milk (drug, water, vitamins, and very occasional formula are permissible while breastfeeding) from birth to the outcome endpoint. In addition, we estimated the average daily formula intake (ml/day) during the period from birth to the outcome endpoint, based on the time at which infant formula addition started, number of feeding times per day, and average intake per feeding during 0–1, 1–3, and 3–6 months. We classified daily formula intake into four levels: 0–250, 250–499, 500–749, and ≥ 750 ml/day.

### 2.5 Covariates

Covariates included were the baseline sociodemographic characteristics and potential confounders (Rajappan et al., 2017; Shi et al., 2015; Simoes, 2003). The baseline sociodemographic variables included maternal education level (0–9, 10–12, and > 12 years according to completed schooling years), household income (0–4,999, 5,000–9,999, and ≥ 10,000 China yuan per month), parental age at delivery, and gestational age. Potential confounders considered were maternal pre‐pregnancy body mass index (BMI, weight [kg]/[height^2^ {m^2^}]; Rajappan et al., 2017), season of birth (spring: March–May; summer: June–September; fall: September–November; and winter: December–February), having infant siblings, parental smoking before pregnancy, infant sex, and infant birthweight (Shi et al., 2015; Simoes, 2003).

### 2.6 Statistical analysis

We performed descriptive statistics to describe the study populations, with continuous variables being reported as mean (*SD*) and categorical variables as number and per cent. Group differences in variables were compared using the Student *t* test or analysis of variance for continuous variables and chi‐square test for categorical variables. Kaplan–Meier survival analysis was used to assess the relationship between vitamin D supplementation and time to the first episode of RTI, and significance was determined by comparing the plotted survival curves of the two groups using the log‐rank test. RRs (95% CIs) were analysed by modified Poisson regression (Zou, 2004) to assess the associations of vitamin D supplementation with risk of RTI, LRTI, and RTI‐related hospitalization, with no covariates adjusted in Model 1; Model 2 adjusted for the variables with significant difference among the vitamin D supplementation groups including maternal education, household income, having infant siblings, and infant formula intake; Model 3 further adjusted for other potential risk confounders including infant sex, season of birth, parental smoking before pregnancy, birthweight (Shi et al., 2015; Simoes, 2003), and maternal prepregnancy BMI (Rajappan et al., 2017). *p* values for trend were determined by a regression model analysis by treating the categories of vitamin D supplementation as a continuous variable (Milton et al., 2018). Considering the vitamin D content difference between human milk and fortified formula, all main analyses were performed first in all infants and then in the feeding subgroups to determine whether the associations vary by feeding.

To investigate the sensitivity of the results, we first conducted the subgroup analysis stratified by potential risk variables, such as season of birth and infant sex. Second, we restricted the analysis to infants with no siblings and whose parents had no smoking history. Statistical analyses were performed using a SAS software program (SAS software Package, Version 9.4; SAS Institute Inc., Cary, NC, USA). All *p* values were two‐sided, and *p* value < .05 was considered statistically significant.

### 2.7 Ethical considerations

All procedures performed in these studies involving human participants were approved by the ethics review committee of Tongji Medical College of Huazhong University of Science and Technology. All participants provided written informed consent upon enrolment.

## 3 RESULTS

### 3.1 Characteristics of the study population

A total of 5,099 women were screened for eligibility, of whom 2,344 attended follow‐up interview at 6 months of infant age. After exclusions, 2,244 mother–infant pairs were selected for inclusion in the final analysis (Figure S1). Except for the maternal education, gestational age, infant birthweight, and season of birth, no other differences in baseline characteristics were observed between the included participants and those excluded (Table S1).

The characteristics of the study population stratified by vitamin D supplementation are summarized in Table 1. Among the 2,244 included mother–infant pairs, the mean maternal age at delivery was 29.2 (±3.5) years and paternal age at delivery was 31.0 (±4.3) years; 1,207 (53.8%) were male infants, and 1,124 (50.1%) were born in autumn or winter. A total of 999 (44.5%) infants had RTI during the first 6 months after birth, of whom 215 developed into LRTI and 126 were hospitalized for RTI.

**Table 1:** Characteristics of study population by vitamin D supplementation

Characteristics, no. (%), or mean (*SD*)^a^		Vitamin D supplementation (days/week)				*p* value
	Overall (*n* = 2,244)	0 (*n* = 307)	1–2 (*n* = 278)	3–4 (*n* = 587)	5–7 (*n* = 1,072)	
Parents and household						
Prepregnancy BMI, kg/m^2^	20.8 (2.7)	20.9 (2.8)	20.8 (2.6)	20.9 (2.8)	20.8 (2.7)	.774
Maternal age at delivery, years	29.2 (3.5)	28.9 (3.6)	29.1 (3.7)	29.1 (3.5)	29.3 (3.5)	.360
Paternal age at delivery, years	31.0 (4.3)	30.6 (4.3)	31.0 (4.5)	31.0 (4.0)	31.0 (4.4)	.343
Maternal education, years						<.001
0–9	304 (13.6)	58 (18.9)	51 (18.4)	81 (13.8)	114 (10.6)	
10–12	592 (26.4)	97 (31.6)	84 (30.2)	155 (26.4)	256 (23.9)	
> 12	1,281 (57.1)	144 (46.9)	133 (47.8)	334 (56.9)	670 (62.5)	
Unknown	67 (3.0)	8 (2.6)	10 (3.6)	17 (2.9)	32 (3.0)	
Household income, ¥						<.001
0–4,999	843 (37.6)	151 (49.2)	102 (36.7)	226 (38.5)	364 (34.0)	
5,000–9,999	932 (41.5)	98 (31.9)	122 (43.9)	248 (42.3)	464 (43.3)	
≥ 10,000	427 (19.0)	50 (16.3)	50 (18.0)	101 (17.2)	226 (21.1)	
Unknown	42 (1.9)	8 (2.6)	4 (1.4)	12 (2.0)	18 (1.7)	
Maternal smoking, yes	72 (3.2)	7 (2.3)	9 (3.2)	20 (3.4)	36 (3.4)	.800
Paternal smoking, yes	760 (33.9)	114 (37.1)	102 (37.0)	191 (32.5)	353 (32.9)	.343
Gestational age, weeks	39.7 (1.1)	39.6 (1.1)	39.6 (1.0)	39.7 (1.1)	39.7 (1.1)	.399
Infants						
Birthweight, g	3,340 (333)	3,356 (327)	3,318 (348)	3,338 (322)	3,343 (336)	.570
Infant sex, male	1,207 (53.8)	168 (54.7)	151 (54.3)	321 (54.7)	567 (52.9)	.879
Siblings, yes	376 (16.8)	64 (20.9)	58 (20.9)	96 (16.4)	158 (14.7)	.017
Season of birth, autumn or winter	1,124 (50.1)	174 (56.7)	132 (47.5)	297 (50.6)	521 (48.6)	.069
Formula intake, ml/day						<.001
0–249	1,565 (69.8)	197 (64.2)	163 (58.6)	369 (62.9)	836 (78.0)	
250–499	234 (10.4)	29 (9.5)	35 (12.6)	77 (13.1)	93 (8.7)	
499–749	260 (11.6)	42 (13.7)	42 (15.1)	84 (14.3)	92 (8.6)	
750–1,105	185 (8.2)	39 (12.7)	38 (13.7)	57 (9.7)	51 (4.8)	
Full breastfeeding, yes	1,128 (50.3)	149 (48.5)	121 (43.5)	254 (43.3)	604 (56.3)	<.001
Outcome variables						
All RTI, yes	999 (44.5)	236 (76.9)	129 (46.4)	259 (44.1)	375 (35.0)	<.001
LRTI, yes	215 (9.6)	88 (28.7)	23 (8.3)	51 (8.7)	53 (4.9)	<.001
RTI‐related hospitalization, yes	126 (5.6)	50 (16.3)	14 (5.0)	31 (5.3)	31 (2.9)	<.001

Abbreviations: BMI, body mass index; LRTI, lower respiratory tract infection; RTI, respiratory tract infection; *SD*, standard deviation.

a Values are means (*SD*) for continuous variables and no. (%) for categorical variables.

Regarding formula intake before the first RTI, most infants (69.8%, *n* = 1,565) had a low intake of less than 250 ml/day, 234 (10.4%) infants consumed 250–499 ml/day, 260 (11.6%) infants consumed 500–749 ml/day, and only 185 (8.2%) infants consumed more than 750 ml/day. There was a total of 1,128 (50.3%) infants fully breastfed before the first RTI. Overall, 307 (13.7%) infants did not receive vitamin D supplementation in the first 6 months of life, 278 (12.4%) received supplements 1–2 days/week, 587 (26.1%) infants received supplements 3–4 days/week, and most infants (47.8%, *n* = 1072) received supplements 5–7 days/week.

Compared with the mothers in group without supplementation, the mothers in the vitamin D supplementation groups were more likely to have higher education level and household income, and infants were less likely to have siblings and had a higher level of formula intake. The rate of RTI was 76.9% in the group without supplementation, 46.4% in the 1–2 days/week supplementation group, 44.1% in the 3–4 days/week supplementation group, and 35.0% in the 5–7 days/week supplementation group (*p* < .001); the LRTI rate in those groups was 28.7%, 8.3%, 8.7%, and 4.9%, respectively (*p* < .001); the RTI‐related hospitalization rate was 16.3%, 5.0%, 5.3%, and 2.9%, respectively (*p* < .001).

### 3.2 Vitamin D supplementation and time to the first RTI episode

**Figure 1:**
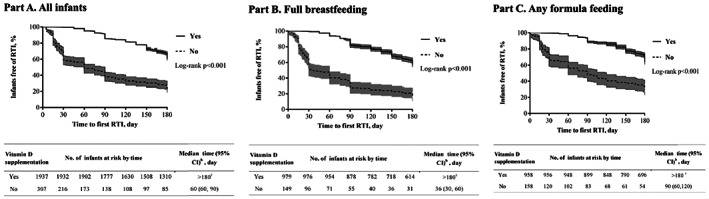
Kaplan–Meier curves^a^ for time to first RTI developed by infants stratified by vitamin D supplementation, according to feeding during the first 6 months of life. The solid line is the Kaplan–Meier curve for the vitamin D supplementation group, and the dotted line for the group without supplementation. ^a^Tinted region indicates 95% confidence intervals around the proportion of infants free of RTI. ^b^Median time indicates the time by which 50% of infants were found experiencing the first RTI episode. ^c^Estimation was not possible because the median time was longer than the 6‐month follow‐up period of the study. Abbreviations: CI, confidence interval; RTI, respiratory tract infection

The time to the first episode of RTI for the vitamin D supplementation groups (yes vs. no) with respect to infant feeding is shown in Figure 1. Infants with vitamin D supplementation developed the first RTI later than those without supplementation in the first 6 months of life, irrespective of their feeding mode (log‐rank *p* value < .001). The median time to their first RTI was 60 days (95% CI [60–90]) for all infants without supplementation, 36 days (95% CI [30–60]) for fully breastfed infants without supplementation, and 90 days (95% CI [60–‐120]) for any formula‐fed infants without supplementation. However, it was not possible to measure the median time to first episode of RTI for infants with vitamin D supplementation, as it was longer than the 6‐month follow‐up study period.

### 3.3 Vitamin D supplementation and susceptibility to RTI

The risk estimates of getting the first RTI by all participants with respect to vitamin D supplementation are given in Table 2, which reveal inverse trends between the frequency of vitamin D supplementation and risk of RTI, LRTI, and RTI‐related hospitalization (*p* for trend < .001 for all groups). Compared with those in the group without supplementation, the unadjusted RRs of RTI were 0.60 (95% CI [0.52, 0.69]) in the 1–2 days/week supplementation group, 0.57 (95% CI [0.51, 0.64]) in the 3–4 days/week supplementation group, and 0.46 (95% CI [0.41, 0.50]) in the 5–7 days/week supplementation group. Meanwhile, the unadjusted RRs of LRTI in those groups were 0.29 (95% CI [0.19, 0.44]), 0.30 (95% CI [0.22, 0.42]), and 0.17 (95% CI [0.13, 0.24]), respectively; the unadjusted RRs of RTI‐related hospitalization were 0.31 (95% CI [0.17, 0.55]), 0.32 (95% CI [0.21, 0.50]), and 0.18 (95% CI [0.12, 0.27]), respectively. All these inverse associations were maintained after adjustment in Model 2 for maternal education, household income, formula consumption, and having siblings. Further adjustment in Model 3 for other potential confounders as infant sex, birthweight, season of birth, maternal prepregnancy BMI, and parental smoking before pregnancy also did not alter the inverse associations.

**Table 2:** Risk ratios and 95% confidence intervals for association between vitamin D supplementation and RTI risk among all infants in the first 6 months of life

RR (95% CI)	Vitamin D supplementation (days/week)				*p* for trend
	0	1–2	3–4	5–7	
All RTI, case/*N*	236/307	129/278	259/587	375/1,072	
Model 1	1	0.60 [0.52, 0.69]	0.57 [0.51, 0.64]	0.46 [0.41, 0.50]	<.001
Model 2	1	0.61 [0.53, 0.70]	0.58 [0.52, 0.65]	0.45 [0.41, 0.50]	<.001
Model 3	1	0.61 [0.53, 0.71]	0.58 [0.53, 0.66]	0.46 [0.41, 0.51]	<.001
LRTI, case/*N*	88/307	23/278	51/587	53/1,072	
Model 1	1	0.29 [0.19, 0.44]	0.30 [0.22, 0.42]	0.17 [0.13, 0.24]	<.001
Model 2	1	0.29 [0.19, 0.45]	0.31 [0.22, 0.42]	0.17 [0.12, 0.24]	<.001
Model 3	1	0.29 [0.19, 0.45]	0.31 [0.22, 0.42]	0.17 [0.12, 0.24]	<.001
RTI‐related hospitalization, case/*N*	50/307	14/278	31/587	31/1,072	
Model 1	1	0.31 [0.17, 0.55]	0.32 [0.21, 0.50]	0.18 [0.12, 0.27]	<.001
Model 2	1	0.32 [0.18, 0.56]	0.32 [0.21, 0.50]	0.17 [0.11, 0.27]	<.001
Model 3	1	0.33 [0.19, 0.59]	0.33 [0.22, 0.51]	0.18 [0.11, 0.28]	<.001

*Note.* Model 1 was unadjusted; Model 2 was adjusted for maternal education level, household income, presence of siblings, and formula intake; Model 3 was further adjusted for other potential factors including infant sex, birthweight, maternal prepregnancy body mass index, parental smoking before pregnancy, and season of birth.

Abbreviations: CI, confidence interval; LRTI, lower respiratory tract infection; RR, risk ratio; RTI, respiratory tract infection.

Similarly, we noticed a reduction in the risk of RTI, LRTI, and RTI‐related hospitalization by vitamin D supplementation in both the full breastfeeding and any formula feeding subgroups, with the lowest risk observed in the 5–7 days/week supplementation group: the unadjusted RRs were 0.44 (95% CI [0.38, 0.50]) for all RTIs; 0.16 (95% CI [0.11, 0.24]) for LRTI and 0.15 (95% CI [0.09, 0.26]) for RTI‐related hospitalization among full breastfed infants (Table 3); and 0.46 (95% CI [0.39, 0.55]) for all RTIs, 0.19 (95% CI [0.11, 0.30]) for LRTI, and 0.21 (95% CI [0.11, 0.43]) for RTI‐related hospitalization, respectively, among any formula‐fed infants. These inverse associations were maintained after adjustments (Table 4).

**Table 3:** Risk ratios and 95% confidence intervals for association between vitamin D supplementation and RTI risk among fully breastfed infants in the first 6 months of life

RR (95% CI)	Vitamin D supplementation (days/week)				*p* for trend
	0	1–2	3–4	5–7	
All RTI, case/*N*	125/149	64/121	130/254	223/604	
Model 1	1	0.63 [0.53, 0.76]	0.61 [0.53, 0.70]	0.44 [0.38, 0.50]	<.001
Model 2	1	0.63 [0.52, 0.76]	0.61 [0.53, 0.70]	0.44 [0.39, 0.50]	<.001
Model 3	1	0.63 [0.52, 0.75]	0.61 [0.53, 0.71]	0.44 [0.39, 0.50]	<.001
LRTI, case/*N*	48/149	10/121	23/254	31/604	
Model 1	1	0.26 [0.14, 0.49]	0.28 [0.18, 0.44]	0.16 [0.11, 0.24]	<.001
Model 2	1	0.25 [0.13, 0.48]	0.28 [0.18, 0.44]	0.16 [0.10, 0.24]	<.001
Model 3	1	0.26 [0.13, 0.49]	0.29 [0.19, 0.46]	0.16 [0.10, 0.25]	<.001
RTI‐related hospitalization, case/*N*	31/149	8/121	12/254	19/604	
Model 1	1	0.32 [0.15, 0.67]	0.23 [0.12, 0.43]	0.15 [0.09, 0.26]	<.001
Model 2	1	0.32 [0.15, 0.67]	0.22 [0.11, 0.43]	0.15 [0.08, 0.26]	<.001
Model 3	1	0.33 [0.16, 0.68]	0.24 [0.13, 0.46]	0.15 [0.09, 0.27]	<.001

*Note.* Model 1 was unadjusted; Model 2 was adjusted for maternal education level, household income, and presence of siblings; Model 3 was further adjusted for other potential confounders including infant sex, birthweight, maternal prepregnancy body mass index, parental smoking before pregnancy, and season of birth.

Abbreviations: CI, confidence interval; LRTI, lower respiratory tract infection; RR, risk ratio; RTI, respiratory tract infection.

**Table 4:** Risk ratios and 95% confidence intervals for association between vitamin D supplementation and RTI risk among any formula‐fed infants in the first 6 months of life

RR (95% CI)	Vitamin D supplementation (days/week)				*p* for trend
	0	1–2	3–4	5–7	
All RTI, case/*N*	111/158	65/157	129/333	152/468	
Model 1	1	0.59 [0.48, 0.73]	0.55 [0.47, 0.62]	0.46 [0.39, 0.55]	<.001
Model 2	1	0.60 [0.48, 0.74]	0.57 [0.48, 0.67]	0.48 [0.40, 0.57]	<.001
Model 3	1	0.61 [0.49, 0.76]	0.56 [0.48, 0.67]	0.48 [0.41, 0.58]	<.001
LRTI, case/*N*	40/158	13/157	28/333	22/468	
Model 1	1	0.32 [0.18, 0.59]	0.33 [0.21, 0.52]	0.19 [0.11, 0.30]	<.001
Model 2	1	0.33 [0.18, 0.59]	0.34 [0.21, 0.54]	0.19 [0.11, 0.32]	<.001
Model 3	1	0.34 [0.19, 0.62]	0.33 [0.21, 0.53]	0.19 [0.12, 0.33]	<.001
RTI‐related hospitalization, case/*N*	19/158	6/157	19/333	12/468	
Model 1	1	0.32 [0.13, 0.78]	0.47 [0.26, 0.87]	0.21 [0.11, 0.43]	<.001
Model 2	1	0.33 [0.13, 0.79]	0.48 [0.25, 0.90]	0.22 [0.10, 0.46]	<.001
Model 3	1	0.36 [0.15, 0.87]	0.49 [0.26, 0.91]	0.23 [0.11, 0.47]	<.001

*Note.* Model 1 was unadjusted; Model 2 was adjusted for maternal education level, household income, presence of siblings, and formula intake; Model 3 was further adjusted for other potential confounders including infant sex, birthweight, maternal prepregnancy body mass index, parental smoking before pregnancy, and season of birth.

Abbreviations: CI, confidence interval; LRTI, lower respiratory tract infection; RR, risk ratio; RTI, respiratory tract infection.

### 3.4 Sensitivity analysis

Sensitivity analysis was performed to examine the robustness of our results. We observed consistent associations of vitamin D supplementation with a lower risk of all RTIs in a subgroup analysis stratified by season of birth and infant sex, with the lowest risk in the 5–7 days/week supplementation for each subgroup. Sensitivity analysis by birth season revealed that the RRs (95% CI) of RTI in the 5–7 days/week supplementation group were 0.44 (0.37–0.52) for infants born in autumn and winter with full breastfeeding, 0.44 (0.37–0.53) for those born in spring and summer with full breastfeeding, 0.40 (0.32–0.51) for infants born in autumn and winter with formula feeding, and 0.55 (0.42–0.71) for those born in spring and summer with formula feeding (Figure S2). The results were significant and similar in sensitivity analysis by infant sex (Figure S3). Further analysis restricted to infants with no siblings and whose parents had no smoking history also remained consistent with the main analysis (Figure S4).

## 4 DISCUSSION

This study provides novel information on the association between vitamin D supplementation and RTI risk during early infancy. We observed a longer period without experiencing the first RTI episode in the first 6 months of life in infants with vitamin D supplementation than in those without supplementation. In addition, vitamin D supplementation to infants was found to be associated with a decreased risk of RTI, LRTI, and RTI‐related hospitalization, showing the greatest risk reduction in the 5–7 days/week supplementation group, irrespective of infant feeding. These results did not vary after adjustment for potential risk confounders. Furthermore, the results of the sensitivity analysis stratified by infant sex, season of birth, and analysis restricted to infants without siblings and whose parents had no smoking history were consistent with main analysis, strongly suggesting the protective effect of vitamin D supplementation against RTI during the first 6 months of life.

Emerging observational studies have found a link between low blood 25(OH)D levels and an increased risk of RTI in the general population and mother–infant group (Binks et al., 2016; Camargo et al., 2011; Magnus et al., 2013; Mohamed & Al‐Shehri, 2012; Monlezun, Bittner, Christopher, Camargo, & Quraishi, 2015; Mulrennan et al., 2018). Given the high susceptibility to RTI in infants (Nair et al., 2013), the effects of vitamin D supplementation on the susceptibility of infants to RTI warrant further investigation. However, only two studies to date have been conducted in infant population and reported inconsistent results (Grant et al., 2015; Manaseki‐Holland et al., 2012). One of them was a small trial that found a significantly reduced incidence of primary care visit for RTI in infants with a daily dose of 800 IU of vitamin D supplementation from birth to 6 months compared with the placebo group (Grant et al., 2015). The other one was a large‐scale trial, different in design and outcome measures from a small trial (Grant et al., 2015), which found no benefit toward pneumonia prevention in infants with a quarterly dose of 100,000 IU of vitamin D supplementation during an 18‐month intervention (Manaseki‐Holland et al., 2012). In children populations, trials have found a beneficial effect of vitamin D supplementation against RTI in those with vitamin D deficiency or insufficiency at baseline (Camargo et al., 2012; Loeb et al., 2019). Such discrepancies may be, at least partially, explained by the heterogeneity of the data included in the studies—for example, sample size of trial, age of subjects, dose of vitamin D supplementation, frequency of vitamin D administration, and baseline vitamin D status of the subjects. Considering these potential effect modifiers, a large‐scale systematic meta‐analysis revealed a benefit of vitamin D supplementation in RTI prevention in the general population, with a stronger protective effect obtained with daily or weekly vitamin D supplementation than with a bolus dose in subjects with severe vitamin D deficiency at baseline (Martineau et al., 2017). In this prospective cohort study, we observed a longer period without the occurrence of the first RTI episode and a lower risk for RTI, LRTI, and RTI‐related hospitalization in infants with vitamin D supplementation than in those without, suggesting a strong protective role for vitamin D supplementation in RTI prevention during infancy.

Given the substantial contribution of infant formula to vitamin D intake due to fortification, we performed subgroup analysis to ascertain whether the inverse association between vitamin D supplementation and RTI susceptibility varied with infant feeding. It should be emphasized that we found a consistent association between vitamin D supplementation and RTI risk among any formula‐fed infants as well as fully breastfed infants. Accordingly, it raised the question of whether increasing the routine vitamin D supplementation dose would be more beneficial for RTI prevention in infancy. Gallo et al. reported that 33% of infants achieved the optimal 25(OH)D of 75 nmol/L or greater (Pludowski et al., 2018) in the 400 IU/day supplementation group, 81% in the 800 IU/day group, 92% in the 1,200 IU/day group, and 100% in the 1,600 IU/day group (Gallo et al., 2013). As the tolerable upper intake levels for infants aged 0 to 6 is 1,000 IU/day according to the Institute of Medicine (Ross et al., 2011), we suggest supplementation of 800 or 1,000 IU/day as two optional daily doses in future studies, aiming at establishing recommendations for vitamin D supplementation for the purpose of preventing RTIs during early infancy.

The biological functions of vitamin D are mainly mediated through the vitamin D receptor, which are widely distributed throughout human tissues (Christakos et al., 2013). The presence of 25(OH)D has been associated with the development of adaptive and innate immune responses, including up‐regulating antimicrobial peptide production, modulating immune cell proliferation, maturation, and cytokine expression, in the first 6 months of life (Jones et al., 2015; Tahamtan, Askari, Bont, & Salimi, 2019; Wei & Christakos, 2015). Also, infants with lower cord serum 25(OH)D levels are prone to relatively poor lung function (Lai et al., 2017), which may explain the mechanism for the involvement of vitamin D in the susceptibility to RTI episode.

To the best of our knowledge, this is the first study to examine the association between routine vitamin D supplementation in infants and infantstheir susceptibility to RTI in a large‐scale prospective birth cohort. Vitamin D supplementation was purposefully assessed from birth to the first episode of RTI in the first 6 months of life, allowing us to gain a prospective insight into the exposure–response association between vitamin D supplementation and RTI risk with less bias. In addition, to eliminate the potential difference of vitamin D intake between human milk and infant formula, we performed all main analyses in fully breastfed infants and any formula‐fed infants, taking the formula intake into account, and the results were consistent in these feeding subgroups. Furthermore, sensitivity analysis stratified by potential risk confounders, such as infant sex and birth season, and analysis restricted to subjects without siblings and whose parents had no smoking history both remained robust with the main analysis, indicating high reliability of our findings.

This study has some potential limitations. First, the mother‐reported data were a limitation in this study, which could potentially be subject to recall bias. Second, a portion of mild RTI episodes that did not result in a visit to the paediatrician may have not been captured, although LRTI and RTI‐related hospitalization were identified as two additional outcomes, which may be valid as an assessment of the true status for severe RTI case. Third, the vitamin D status at the baseline was not measured in this study. Neonate was likely to be born with vitamin D insufficiency or deficiency due to the high prevalence of vitamin D deficiency in pregnancy (Song et al., 2013; Zhao et al., 2017); thus, the protective effect of routine vitamin D supplementation on RTI might be attributed to the replacement of vitamin D. However, we were unable to show whether vitamin D supplementation would bring a similar benefit to those with sufficient vitamin D status. In addition, we were unable to fully adjust the potential risk factors, including the numbers of people in the house and immunization status. Lastly, as an observational study, it might not be adequate to make well‐founded causal inferences; thus, we expect future randomized control trials to further confirm our findings, taking these limitations into account.

In conclusion, routine vitamin D supplementation to infants was associated with a decreased risk of RTI, LRTI, and RTI‐related hospitalization, in the first 6 months of life, irrespective of infant feeding. Our findings provide new evidence of the promising preventative effect of routine vitamin D supplementation against RTI during early infancy. Moreover, the present study further highlights the importance of a higher dose of vitamin D supplementation in future study design to determine the optimal recommendation for routine vitamin D supplementation in the prevention of RTI.

## CONFLICTS OF INTEREST

The authors declare that they have no conflicts of interest.

## CONTRIBUTIONS

HM collected data, performed the data analyses, and drafted the manuscript. XT, HJ, WY, LL, ZZ, HL, WH, and KC participated in data collection and validation and helped review and edit the manuscript. HL, YN, and YX conceptualized and designed the study, coordinated and supervised data collection, and critically reviewed the manuscript for important intellectual content. All authors gave final approval of the work to be published and agreed to be accountable for all aspects of the work.

## Supporting information


**Figure S1**. Flow chart

**Figure S2**. Subgroup analysis of the association between vitamin D supplementation and respiratory tract infection, stratified by feeding and birth season

**Figure S3**. Subgroup analysis of the association between vitamin D supplementation and respiratory tract infection, stratified by feeding and gender

**Figure S4**. Subgroup analysis of the association between vitamin D supplementation and respiratory tract infection, restricted to infants without siblings and whose parents had no smoking history

**Table S1.** Baseline characteristics of mother–infant pairs between the included and excluded groups
