# Supplementary figures and images for: Association of vitamin D supplementation with respiratory tract infection in infants
Source: Matern Child Nutr. 2020 Mar 5;16(3):e12987. doi: 10.1111/mcn.12987 (PMC7296792; doi:10.1111/mcn.12987)

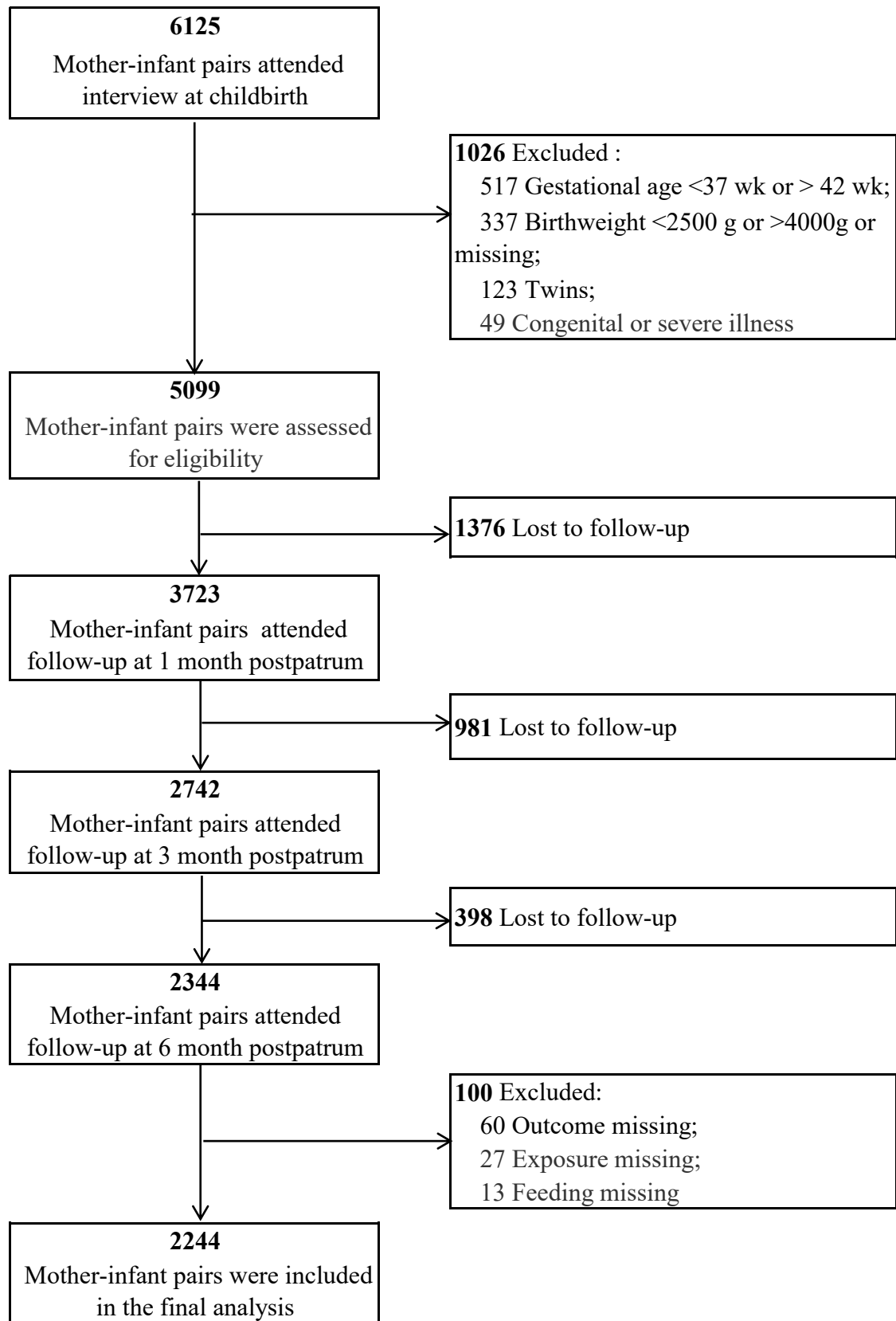

**Supplementary Figure 1.** Flow chart

Supplement: Supplementary file 1 — Figure S1. Flow chart [file MCN-16-e12987-s001.pdf]
