# Supplementary material for: Association of vitamin D supplementation with respiratory tract infection in infants
Source: Matern Child Nutr. 2020 Mar 5;16(3):e12987. doi: 10.1111/mcn.12987 (PMC7296792; doi:10.1111/mcn.12987)
Supplement: Supplementary file 2 — Figure S2. Subgroup analysis of the association between vitamin D supplementation and respiratory tract infection, stratified by feeding and birth season [file MCN-16-e12987-s002.pdf]

Subgroubs	Case/N	Risk Ratios (95% CIs)	*P* Value
**Full breastfeeding, Autumn and Winter**			
Vitamin D supplementation (days/ week)			
0	69/81	1	
1-2	35/64	0.64 (0.50-0.82)	<0.001
3-4	68/135	0.59 (0.49-0.72)	<0.001
5-7	106/284	0.44 (0.37-0.52)	<0.001
**Full breastfeeding, Spring and Summer**			
Vitamin D supplementation (days/ week)			
0	56/68	1	
1-2	29/57	0.62 (0.47-0.82)	<0.001
3-4	62/119	0.63 (0.52-0.78)	<0.001
5-7	117/320	0.44 (0.37-0.53)	<0.001
**Any formula feeding, Autumn and Winter**			
Vitamin D supplementation (days/ week)			
0	70/93	1	
1-2	32/68	0.63 (0.47-0.83)	<0.001
3-4	66/162	0.54 (0.43-0.67)	<0.001
5-7	72/237	0.40 (0.32-0.51)	<0.001
**Any formula feeding, Spring and Summer**			
Vitamin D supplementation (days/ week)			
0	41/65	1	
1-2	33/89	0.59 (0.42-0.82)	<0.001
3-4	63/171	0.58 (0.45-0.77)	<0.001
5-7	80/231	0.55 (0.42-0.71)	0.001

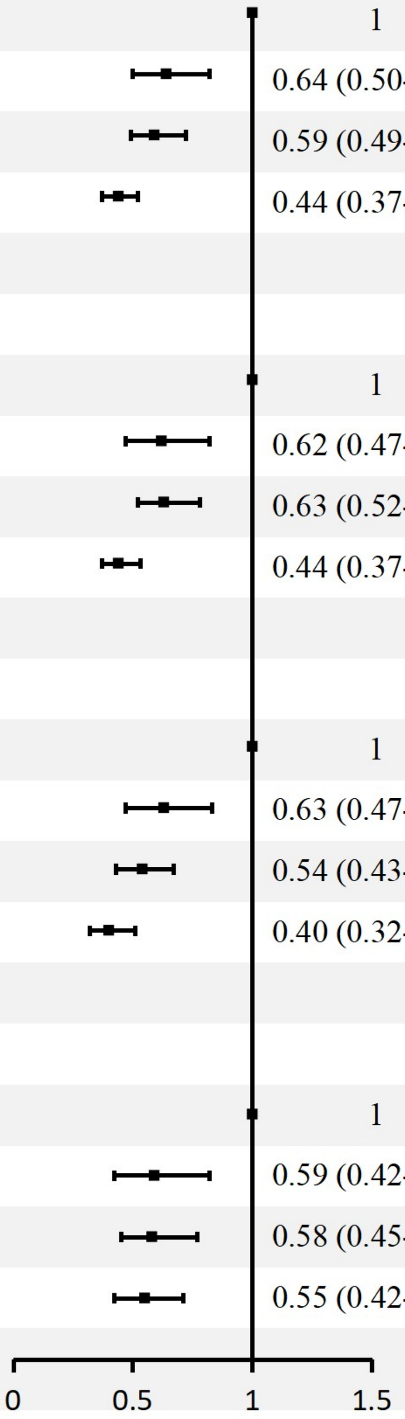

Supplementary Figure 2. Subgroup analysis of the asscociation between vitamin D supplementation and respiratory tract infection, stratified by feeding and birth season
