# Supplementary material for: Association of vitamin D supplementation with respiratory tract infection in infants
Source: Matern Child Nutr. 2020 Mar 5;16(3):e12987. doi: 10.1111/mcn.12987 (PMC7296792; doi:10.1111/mcn.12987)
Supplement: Supplementary file 3 — Figure S3. Subgroup analysis of the association between vitamin D supplementation and respiratory tract infection, stratified by feeding and gender [file MCN-16-e12987-s003.pdf]

Subgroups	Case / N	Risk Ratios (95% CIs)	*P* Value
**Full breastfeeding, boy**			
Vitamin D supplementation (days/ week)			
0	70/80	1	
1-2	42/67	0.72 (0.59-0.88)	0.001
3-4	71/132	0.61 (0.51-0.73)	<0.001
5-7	128/320	0.45 (0.39-0.54)	<0.001
**Full breastfeeding, girl**			
Vitamin D supplementation (days/ week)			
0	55/69	1	
1-2	22/54	0.51 (0.36-0.72)	<0.001
3-4	59/122	0.61 (0.49-0.76)	<0.001
5-7	95/284	0.42 (0.34-0.51)	<0.001
**Any formula feeding, boy**			
Vitamin D supplementation (days/ week)			
0	64/88	1	
1-2	35/84	0.57 (0.43-0.76)	<0.001
3-4	81/189	0.59 (0.48-0.73)	<0.001
5-7	86/247	0.48 (0.39-0.59)	<0.001
**Any formula feeding, girl**			
Vitamin D supplementation (days/ week)			
0	47/70	1	
1-2	30/73	0.61 (0.44-0.84)	0.003
3-4	48/144	0.50 (0.37-0.66)	<0.001
5-7	66/221	0.44 (0.34-0.58)	<0.001

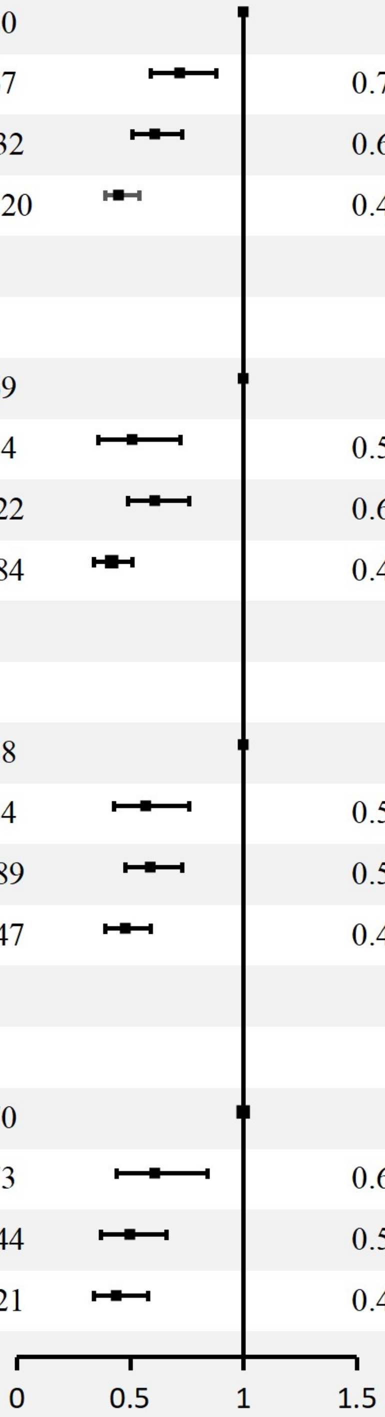

Supplementary Figure 3. Subgroup analysis of the asscociation between vitamin D supplementation and respiratory tract infection, stratified by feeding and gender
