# Supplementary material for: Association of vitamin D supplementation with respiratory tract infection in infants
Source: Matern Child Nutr. 2020 Mar 5;16(3):e12987. doi: 10.1111/mcn.12987 (PMC7296792; doi:10.1111/mcn.12987)
Supplement: Supplementary file 4 — Figure S4. Subgroup analysis of the association between vitamin D supplementation and respiratory tract infection, restricted to infants without siblings and whose parents had no smoking history [file MCN-16-e12987-s004.pdf]

Subgroups	Case / N	Risk Ratios (95% CIs)	*P* Value
**Full breastfeeding**			
Vitamin D supplementation (days/ week)			
0	65/78	1	
1-2	35/62	0.68 (0.53-0.86)	<0.001
3-4	76/165	0.55 (0.46-0.67)	<0.001
5-7	125/352	0.43 (0.36-0.51)	<0.001
**Any formula feeding**			
Vitamin D supplementation (days/ week)			
0	50/74	1	
1-2	28/75	0.55 (0.40-0.77)	<0.001
3-4	60/172	0.52 (0.40-0.67)	<0.001
5-7	76/260	0.43 (0.34-0.55)	<0.001

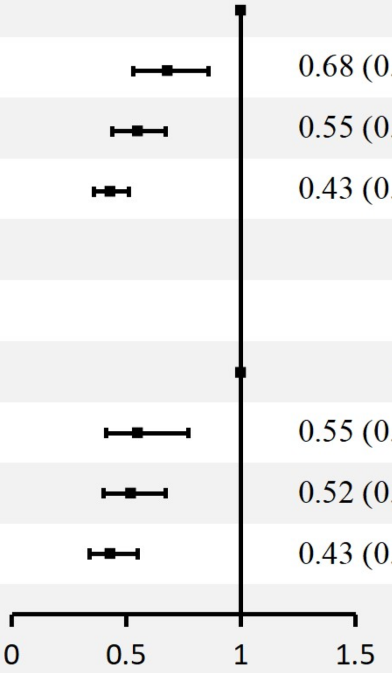

Supplementary Figure 4. Subgroup analysis of the asscociation between vitamin D supplementation and respiratory tract infection, restricted to infants without siblings and whose parents had no smoking history
