# Supplementary material for: Association of vitamin D supplementation with respiratory tract infection in infants
Source: Matern Child Nutr. 2020 Mar 5;16(3):e12987. doi: 10.1111/mcn.12987 (PMC7296792; doi:10.1111/mcn.12987)
Supplement: Supplementary file 5 — Table S1. Baseline characteristics of mother–infant pairs between the included and excluded groups [file MCN-16-e12987-s005.doc]

**Supplementary Table 1.** Baseline characteristics of mother-infant pairs between the included and excluded groups

| **Characteristics,**  **No. (%) or Mean (SD)** *^a^* |  | **Participants** | | |
| --- | --- | --- | --- | --- |
|  |  | **Included**  **(n=2244)** | **Excluded (n=2855)** | ***P* value** |
| Maternal age at delivery, y |  | 29.2 (3.5) | 29.1 (3.5) | 0.550 |
| Paternal age at delivery, y |  | 31.0 (4.3) | 30.9 (4.2) | 0.763 |
| Maternal education, y |  |  |  | 0.032 |
| 0-9 |  | 304 (13.6) | 453 (15.9) |  |
| 10-12 |  | 592 (26.4) | 771 (27.0) |  |
| >12 |  | 1281 (57.1) | 1568 (54.9) |  |
| Unknown |  | 67 (3.0) | 63 (2.2) |  |
| Household income, ¥ |  |  |  |  |
| <5000 |  | 843 (37.6) | 1120 (39.2) | 0.519 |
| 5000~ |  | 932 (41.5) | 1170 (41.0) |  |
| 10000~ |  | 427 (19.0) | 507 (17.8) |  |
| Unknown |  | 42 (1.9) | 58 (2.0) |  |
| Gestational age, weeks |  | 39.7 (1.1) | 39.6 (1.1) | 0.033 |
| Siblings, yes |  | 376 (16.8) | 475 (16.6) | 0.914 |
| Maternal smoking, yes |  | 72 (3.2) | 90 (3.2) | 0.671 |
| Paternal smoking, yes |  | 760 (33.9) | 933 (32.7) | 0.456 |
| Pre-pregnancy BMI, kg/m^2^ |  | 20.8 (2.7) | 20.7 (2.7) | 0.104 |
| Birth weight, g |  | 3340 (333) | 3318 (331) | 0.016 |
| Infant sex, male |  | 1207 (53.8) | 1483 (51.9) | 0.200 |
| Season of birth, autumn or winter |  | 1124 (50.1) | 1583 (55.5) | <0.001 |

Definition of abbreviations: SD, standardized deviation; BMI, body mass index.

*^a^* Values are means (SD) for continuous variables and No. (%) for categorical variables.
